# Real-Life Challenges in Assessing Nutritional Status and Quality of Life in Patients with Cirrhosis

**DOI:** 10.3390/diagnostics15243206

**Published:** 2025-12-15

**Authors:** Ioana Parola, Ilinca Savulescu-Fiedler, Sandica Bucurica, Ionela Maniu, Bianca Cheaib, Mariana Jinga

**Affiliations:** 1 Department of Gastroenterology, “Carol Davila” University of Medicine and Pharmacy, 020021 Bucharest, Romania; ioana.prodan96@gmail.com (I.P.); mariana.jinga@umfcd.ro (M.J.); 2 Department of Internal Medicine, “Carol Davila” University of Medicine and Pharmacy, 050474 Bucharest, Romania; 3 Department of Internal Medicine, Coltea Clinical Hospital, 030167 Bucharest, Romania; 4 Department of Gastroenterology, “Dr. Carol Davila” Central Military Emergency University Hospital, 010825 Bucharest, Romania; 5 Department of Mathematics and Informatics, Faculty of Sciences, Lucian Blaga University Sibiu, 550012 Sibiu, Romania; ionela.maniu@ulbsibiu.ro; 6 Research Team, Pediatric Clinical Hospital Sibiu, 550166 Sibiu, Romania; 7 Department of Medical Oncology, Gustave Roussy Institute, 94805 Villejuif Cedex, France; bianca.cheaib@gustaveroussy.fr

**Keywords:** quality of life, cirrhosis, nutritional status, malnutrition, advanced liver disease

## Abstract

**Background**: Liver cirrhosis is a chronic systemic disease with a prevalence of 1.3% worldwide. Malnutrition refers to an imbalance of essential nutrients or altered utilization, with a prevalence ranging from 5 to 92%. The aim of this study is to assess the quality of life of cirrhotic patients and to investigate the incidence of malnutrition, thereby enabling the identification of high-risk groups by evaluating commonly used nutritional assessment tools in everyday clinical practice and identifying discrepancies between objective and subjective measures in cirrhotic patients. **Methods**: This is a single-center prospective study including patients diagnosed with liver cirrhosis from a tertiary center. **Results**: We included 53 patients, 81.13% (*n* = 43) of whom were men, with a mean age of 62.36 ± 9.28. Most patients had hypoalbuminemia, vitamin D deficiency, and low levels of cholesterol, triglycerides, and magnesium. 64.15% (*n* = 34) had malnutrition according to the RFH-NPT test, while the SGA questionnaire revealed a high predominance of the A class. Higher mean MELD, MELD-Na, and MELD 3.0 scores were associated with higher RFH-NPT and SGA scores. The CLDQ presented lower mean values for disease progression. **Conclusions**: This study is a real-world evaluation of patients with liver cirrhosis referred to a tertiary center, revealing a low quality of life of the patients and a high prevalence of malnutrition.

## 1. Introduction

Liver cirrhosis is a chronic systemic disease with a prevalence of 1.3% worldwide, 500–1100 cases per 100,000 inhabitants in Europe, with increased mortality and morbidity [1,2]. The quality of life of cirrhotic patients is severely affected by numerous complications, including the need for hospitalization, increased risk for depression, muscle cramps, frailty, fatigue, malnutrition, sleep disturbances, altered cognitive function, and inability to work [3,4].

Malnutrition refers to deficiencies or excesses of nutrients, imbalances in essential nutrients, or altered nutrient utilization [5]. The association of malnutrition with disease emphasizes reduced food intake/assimilation and a variable range of acute or chronic inflammation [6]. It has been recognized as a severe burden in liver cirrhosis, needing timely and proper care to improve prognosis. The prevalence of malnutrition in cirrhosis has been evaluated between 5% and 92%, a variability that can be attributed to underdiagnosis and lack of proper evaluation. The progression from a compensated to a decompensated state of cirrhosis is associated with an increase in the proportion of malnourished patients, from 20% to 50% [7,8]. It has been emphasized that there is high prevalence of malnutrition among patients with Child–Pugh C cirrhosis, over 50% [9,10], with one study showing a prevalence of 95% for these patients [11]. Malnutrition has been linked to a low body mass index (BMI) < 18.5 kg/m^2^ and Child–Pugh C [8,12].

Even though screening for malnutrition is difficult due to ascites and peripheral edema, it should be assessed in every cirrhotic patient [7].

Sarcopenia represents another complication that can be found in cirrhotic patients; it is a progressive and generalized muscle disease defined by low levels of muscle strength, quantity, or quality, and low physical performance as a marker for severity [13]. Sarcopenia has been associated with malnutrition, as both involve low muscle mass; however, malnutrition is also defined by low fat mass [13]. The incidence of sarcopenia in cirrhosis has been determined to be 26–34% [14,15,16]; it has been associated with increased risk of infection, lengthy hospitalization, hepatic encephalopathy (HE), poor quality of life (QoL), and higher medical costs [17,18,19].

The aim of this study is to evaluate the quality of life of cirrhotic patients, identify risk factors associated with it, and investigate the incidence of malnutrition and/or sarcopenia, thereby enabling the identification of high-risk groups and the implementation of appropriate measures.

## 2. Materials and Methods

### 2.1. Study Design and Eligibility Criteria

This is a single-center, prospective, observational study. All patients diagnosed with liver cirrhosis admitted to the Central University Emergency Military Hospital, Bucharest, between January 2024 and June 2025 were included in the study. The inclusion criteria were patients with liver cirrhosis, above 18 years, without malignancies or hepatic encephalopathy stage III or IV West Haven.

We excluded patients based on the following criteria:

(1) hepatocellular carcinoma (HCC) or other malignancy,
(2) septicemia,
(3) respiratory insufficiency,
(4) hepatorenal syndrome,
(5) coexisting Human Immunodeficiency Virus or tuberculosis,
(6) inflammatory bowel disease or other malabsorptive disorders,
(7) patients receiving glucocorticoid or immunosuppressive drugs, and
(8) patients who were unable to sign the informed consent or complete the questionnaires.

**Figure 1:**
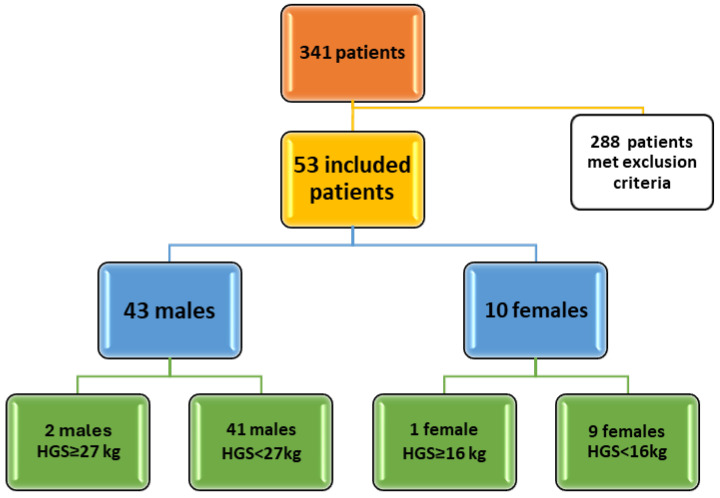
Study flow diagram.

There have been many patients evaluated in the clinic, 341 patients, but due to extensive exclusion criteria, only 53 patients could be included (Figure 1).

This study was conducted in accordance with the Declaration of Helsinki and was approved by the Ethics Committee of Carol Davila Central Military Emergency University Hospital, Bucharest, no. 642/07.11.2023. Informed consent was obtained from all patients.

### 2.2. Clinical and Paraclinical Characteristics

We collected a series of data including age, sex, height, weight, the etiology and stage of cirrhosis, comorbidities (the presence of diabetes mellitus (DM), chronic kidney disease (CKD)), cirrhosis related complications (ascites and the level of it, HE and esophageal varices), peripheric edema, and medication (beta blockers, antisecretory drugs, antibiotics).

The laboratory profile included the hemoglobin and clotting profile, biochemistry-alanine transaminase (ALT), aspartate aminotransferase (AST), gamma-glutamyl transferase (GGT), total bilirubin, albumin, urea, creatinine, cholesterol, triglycerides, ammonia, sodium, potassium, and magnesium levels, B12 vitamin, folic acid, iron, and vitamin D levels.

Patients were classified as underweight if BMI < 18.5 kg/m^2^, normal weight for a BMI between 18.5 and 24.9 kg/m^2^, and overweight if BMI > 25 kg/m^2^ [20]. A correction of the BMI has been applied depending on the grade of ascites and on the presence of peripheric edema −5% for mild ascites, 10% for moderate ascites, 15% for severe, and another 5% for peripheric edema. WHO recommends a BMI < 18.5 kg/m^2^ as a cut-off for diagnosing malnutrition [12].

According to EASL guidelines on nutrition in chronic liver disease, a nutritional screening must be performed in all patients diagnosed with cirrhosis, with proper evaluation for malnutrition [8].

For the evaluation of malnutrition, the Global Leadership Initiative on Malnutrition (GLIM) criteria were used, which include five criteria: non-volitional weight loss; low body mass index; reduced muscle mass; reduced food intake/assimilation; and disease burden/inflammation (Table 1) [6].

The revised European Working Group on Sarcopenia in Older People guidelines (EWGSOP 2), published in 2019, were used for the evaluation of 2 criteria for sarcopenia, low muscle strength and low physical performance [13]. Cut-off points were defined for each method (Table 2). The incidence of sarcopenia could not be evaluated, as not all patients had undergone computer tomography (CT) or dual-energy X-ray absorptiometry (DEXA).

### 2.3. Questionnaires and Other Tests

We used various questionnaires to assess malnutrition and/or sarcopenia and to evaluate subclinical hepatic encephalopathy.

A psychometric test was used to evaluate the patients’ mental status. The Reitan number connection test was applied for every patient to identify subclinical hepatic encephalopathy. The number connection test from the Reitan test involves connecting the printed numbers on the paper from 1 to 25 in the correct order as quickly as possible. The time spent solving the test is the test’s score, including time spent correcting mistakes. A low score indicated good mental status [21,22].

The Chronic Liver Disease Questionnaire (CLDQ) includes 29 items that regard abdominal symptoms, fatigue, systemic symptoms, activity, emotional function, and worry [23]. The responses of this test are from 1 to 7, ranging from “all of the time” to “none of the time,” and it is easy to complete in 10 min [23]. The test was designed to assess health-related quality of life in patients with chronic liver disease [24].

The subjective global assessment (SGA) is a questionnaire that refers to an overall evaluation of a patient’s history and physical assessment, and also uses clinical parameters to diagnose malnutrition [25,26]. This test assesses five factors: changes in weight, dietary intake, gastrointestinal symptoms, functional capacity, and the impact of the disease on nutritional needs; and five physical findings: loss of subcutaneous fat, muscle wasting, ankle edema, sacral edema, and ascites. Based on these, the patients can be classified into three categories: A—well-nourished; B—moderately malnourished; and C—severely malnourished [25]. Guidelines have endorsed SGA as a reliable tool for diagnosing malnutrition in cirrhotic patients and for predicting outcome [27].

The Royal Free Hospital-Nutritional Prioritizing Tool (RFH-NPT) is another test that can assess the nutritional status of cirrhotic patients, validated by the ESPEN guideline [28]. It includes five measurements: BMI, unplanned weight loss, dietary intake, hepatitis severity, and interference with food intake due to current complications (e.g., ascites). It separates patients into three categories: low (score 0), medium (score 1), or high (score 2–7) [29].

Muscle strength was assessed by handgrip strength of the dominant hand using a handgrip dynamometer (Model Saehan^®^, Changwon-si, Republic of Korea). Low grip strength was defined according to the EWGSOP II guidelines [13].

The 6 min walk test (6MWT) was performed on the ward, on a flat, straight 50 m corridor at their usual walking speed. Participants were instructed to walk for 6 min, and the distance was measured afterward. The walking distance was reported for age, height, and weight [30]. A gait speed < 0.8 m/s was considered diagnostic for reduced physical activity [13].

### 2.4. Statistical Analysis

Descriptive analyses were used for general characteristics, and continuous variables are reported as medians and interquartile range (IQR: percentile 25–percentile 75). Categorical data are expressed as numbers (*n*) and proportions (%). A *p*-value < 0.05 was considered significant. The diagnostic accuracy was assessed with the area under the curve (AUC) analysis of receiver operating characteristic (ROC) curves. Spearman and Pearson correlations were used to assess the relationship between variables. Statistical analyses were performed using IBM SPSS version 26 and R v.4.0.5 software.

## 3. Results

### 3.1. Baseline Characteristics

We included 53 cirrhotic patients who met all inclusion criteria, of whom 81.13% (*n* = 43) were men. The mean age was 62.36 ± 9.28, with a younger mean for men. The main etiology was related to alcohol abuse, 75.47% of the group, with an approximately equal distribution between Child–Pugh stages (A-30.2% vs. B-43.4% vs. C-26.4%). The mean for MELD, MELD-Na, and MELD 3.0 scores were 15.67, 18.01, and 18.30 points, respectively (Table 3). 29.09% of patients presented with grade 3 ascites, and 52.72% presented with peripheral edema. The BMI and corrected BMI were included to ensure an accurate evaluation of the patients’ nutritional status, which showed that more than half of the patients were overweight, despite percentage adjustments for ascites and peripheral edema.

The laboratory profile showed that most of the patients had hypoalbuminemia, vitamin D deficiency, low levels of cholesterol, triglycerides, and magnesium, and hyperammonemia. Moreover, an inflammatory status was observed in most of the patients by elevated PCR. Elevation in the renal function, the coagulation profile, bilirubin, sodium, and lower folic acid levels were observed in a subset of patients (Table 3).

### 3.2. The Quality of Life of Cirrhotic Patients

**Figure 2:**
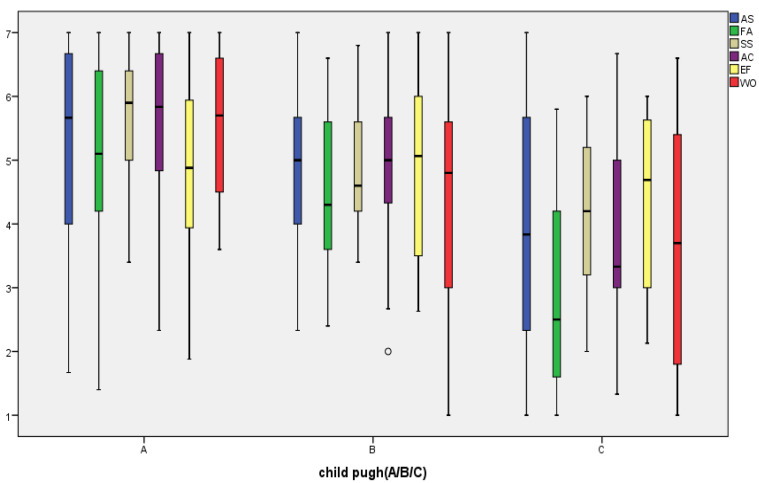
Distribution of CLDQ score/sub-scores according to disease severity evaluated with Child–Pugh Score. AS = abdominal symptoms, FA = fatigue, SS = systemic symptoms, AC = activity, EF = emotional function, WO = worry.

Mean scores of the CLDQ varied between 4.26 and 4.95, with an overall reliability of 0.854, while the reliability for all domains was above 0.795 (Table 4). According to the MELD, MELD-Na, and MELD 3.0 scores, there is a fair correlation between the fatigue and systemic symptoms domains of the CLDQ and the severity of cirrhosis, while with the Child–Pugh score, there is a moderate correlation with fatigue and a fair correlation with systemic symptoms, activity, and worry. Evaluating the mean of the CLDQ domains according to the Child–Pugh score, lower mean values were observed for patients with Child–Pugh C in all sub-scores, with two domains without significant difference—abdominal symptoms and emotional function—emphasizing a poor quality of life with the progression of liver cirrhosis (r= −0.372, *p* < 0.05) (Table 4 and Table 5, Figure 2).

### 3.3. Malnutrition and Sarcopenia Evaluation

The subclinical hepatic encephalopathy was evaluated using the Reitan test, revealing a high incidence in the studied group, 43.39% (*n* = 23).

According to the GLIM criteria, 39.62% (*n* = 21) of the patients were malnourished, while using the RFH-NPT test, 64.15% (*n* = 34) had malnutrition (49.05%—high risk of malnutrition; 15.09%—medium risk to malnourished). The RFH-NPT has a high sensitivity—71.4%—and high specificity—65.6%—with a positive predictive value of 57.7% and an area under the curve of 0.751 for identifying patients at high risk of malnutrition according to the GLIM criteria. Furthermore, RFH-NPT score is associated with the CLDQ (*p* = 0.010), showing a significant relationship in almost all domains, except for abdominal symptoms.

On the other hand, the SGA questionnaire showed a high predominance of the A class at 66.03% (*n* = 35) of the patients (Table 6). No association was found between SGA and the quality of life of the cirrhotic patients evaluated with the CLDQ (*p* = 0.136). However, the fatigue domain showed a significant association with the SGA (*p* = 0.018).

Almost half of the patients with a high risk at the RFH-NPT test were in Child–Pugh B or C. 54.71% of the patients with SGA-A were in Child–Pugh A or B class, while the others were approximately equally distributed (Table 6).

Higher mean MELD, MELD-Na, and MELD 3.0 scores were observed with the rise in the RFH-NPT and SGA scores, with MELD-Na and MELD 3.0 values of more than 20 points for SGA B and C and RFH-NPT-high (Table 6).

HGS revealed that the mean was 15.96 ± 6.81 kg for all groups: 17.49 ± 1.00 kg for men and 9.40 ± 0.83 kg for women. Moreover, all of the women had low muscle strength, HGS < 16 kg according to the cut-off values of EWGSOP II, while, in the male group, it was found to be 77.36% (*n* = 41) of the patients (*p* < 0.005) (Table 3).

The physical performance for evaluating patients showed that 50.94% (*n* = 27) had a gait speed < 0.8 m/s (21 males and 6 females), emphasizing that they might be considered to have severe sarcopenia.

## 4. Discussion

This study presents an in-depth evaluation of the quality of life and nutritional status of patients with liver cirrhosis, most of them with different grades of ascites, revealing a low quality of life in the studied group, a high incidence of malnutrition, and many nutritional deficiencies.

Our findings add to the growing body of evidence that malnutrition is highly prevalent among patients with cirrhosis, although the exact rates depend markedly on both the population studied and the assessment method used. In our cohort, using GLIM criteria, 39.62% of patients were malnourished; by contrast, the RFH-NPT flagged 64.15% of patients as malnourished or at nutritional risk. This variation underlines the well-described heterogeneity in malnutrition prevalence in cirrhosis: previous reports have ranged from approximately 5% to over 90%, depending on tools, settings, and disease severity [31,32,33].

The high incidence of cirrhosis related to alcohol abuse has already been linked as a significant predictor for malnutrition and sarcopenia [33,34,35,36,37].

It has been shown that hospitalized patients have lower BMI and mid-upper arm circumference, higher frailty, and reduced energy and protein intake than outpatients [38]. Moreover, women show greater protein deficits than men, and a higher prevalence of sarcopenia, emphasizing the importance of sex-related nutritional approaches [38]. Furthermore, low BMI, high Child–Pugh, MELD, and MELD-Na scores, and protein and energy deficits were independent predictors of hospitalization [38].

A German study, including 1985 patients, revealed that malnutrition and TIPS are predictors for falls in hospitalized patients, while in-hospital mortality is associated with falling during hospitalization, high MELD score, and infections [39]. Furthermore, another study identified that 45.29% of patients were malnourished, with a high risk of complications (89.61% vs. 39.78%). The sodium and hemoglobin levels were found to be contributing factors to the nutritional status [40].

Regarding malnutrition, the following risk factors have been found: male gender, Child–Pugh C score, hypoalbuminemia, inflammation, low protein intake, vitamin D deficiency, alcohol abuse, hepatic encephalopathy, ascites, and reduced lipid absorption [7,41,42,43].

A Romanian study identified that high Child–Pugh and MELD-Na scores, hyperbilirubinemia, and the presence of spontaneous bacterial peritonitis (SBP) are associated with worse clinical outcomes. Cirrhotic patients with Child–Pugh class C have been found to have a 3.5-fold-greater risk of malnutrition compared to those in classes A or B, and male patients have a 3.4 times higher risk than women [41]. Furthermore, mortality was linked to hypoalbuminemia, hyponatremia, thrombocytopenia, and high PCR [44].

The BMI and corrected BMI identified a low percentage of malnourished cirrhotic patients (5.66% vs. 11.32%), which has low relevance. Similar data have been reported [41,45,46]. Topan et al. also showed that after adjusting for BMI, 54.4% of the patients were overweight [41]. This finding is consistent with the present research; however, it should be interpreted with caution, and an assessment of sarcopenia is necessary, as some patients may have sarcopenic obesity.

A study evaluating cirrhotic patients on the transplant list emphasized that a 6MWD under 250 m represents a marker for severe frailty [47]. Furthermore, a walking distance under 410 m is associated with a 4-fold-higher risk of negative outcomes [48], while Henrique et al. pointed out that a distance under 401.8 m is associated with a decompensation-free outcome rate of 30% [30]. Our results show a lower mean distance in the 6MWD (246.41 ± 155.77), which can be interpreted in the context of decompensated disease, particularly in the presence of ascites.

In this research, the CLDQ for assessing the QoL of patients demonstrated acceptable internal consistency (Cronbach’s alpha = 0.854) and mean domain scores ranging from 4.26 to 4.95. Moreover, the mean CLDQ domain scores were lower in Child–Pugh C patients. The mean values of all domains were lower than those reported in many studies for patients with liver cirrhosis [24,49,50,51,52], revealing a low quality of life for the patients. On the other hand, a Romanian study from 2021 reported mean domain values between 3.77 and 4.68 with a high reliability of 0.93, with a decreasing score as the disease progresses [53]. Another European study evaluating the usefulness of the CLDQ in the Serbian population reported a mean of 4.62 ± 1.11 and a Cronbach’s alpha of 0.93 for the total CLDQ. Moreover, cirrhotic patients with Child–Pugh C had lower sub-scores for abdominal symptoms, fatigue, systemic symptoms, and worry domains [54]. This aspect aligns with our findings, except for a minor exception regarding abdominal symptoms. The severity of liver cirrhosis, as assessed by Child–Pugh scores, MELD, MELD-Na, and MELD 3.0, correlates with worsening CLDQ. Taru et al. validated the effectiveness of the CLDQ in the Romanian population in 2021 on 230 patients, showing an excellent reliability (Cronbach’s alpha 0.93) and a relationship with clinical deterioration, mostly related to ascites [53].

Even though in our study there is a slight difference between the number of malnourished patients identified with RFH-NPT and GLIM criteria, its incidence is similar to those reported in different studies [29,41,55]. RFH-NPT was found to be an important screening method for malnutrition in a study on 166 cirrhotic patients (52.4% identified with RFH-NPT vs. 57.3% using GLIM criteria) (sensitivity (80%), specificity (79%), ROC curve of 0.823) [55]. RFH-NPT has identified faster malnourished patients with Child–Pugh Score B and C, and it represents an independent mortality predictor [29,41]. Another study involving 363 cirrhotic patients that applied the GLIM criteria for malnutrition and the RFH-NPT screening tool reported a 36.4% prevalence of malnutrition when both methods were used. Additionally, the most common GLIM etiologic–phenotypic association was low BMI paired with inflammation [56].

Moreover, RFH-NPT has been found to be a valuable tool for assessing nutritional status in cirrhotic patients. It has been used to predict patient outcomes using a nomogram. Malnutrition is highly prevalent among non-survivors (81.1%), with a good accuracy in predicting survival at 1, 3, and 5 years [57]. On the other hand, RFH-NPT represents an important predictor for low QoL of patients with liver cirrhosis, having an inverse correlation with CLDQ, even after adjustment for age, BMI, and markers of decompensation [58,59].

The SGA performance for evaluating malnutrition has shown variable incidence, 46–66% [27,45,60,61]. A Romanian study, published in 2022, showed that 64.7% of cirrhotic patients were malnourished [41]. Moreover, a negative correlation between SGA score and quality of life of cirrhotic patients was observed [62,63]. In a Japanese study of 345 cirrhotic patients, the majority were categorized as SGA-A (54%), followed by SGA-B (34%) and almost double that of SGA-C (14%), in accordance with our findings. Moreover, SGA-B and SGA-C were independently associated with malnutrition and were mortality predictors. [27]. However, a small Turkish study on 30 patients showed that most of the patients were in the SGA-B, 53.3%, but with nutritional counseling, most of them were rescreened in the A class (60% vs. 36.7% in the B class) [64].

While subjective assessments such as SGA retain a prognostic value (including links to survival) in cirrhosis, they may under-detect muscle depletion or volume loss masked by ascites or edema [10,27]. The discrepancies of our findings between SGA, which showed a predominance of class A patients, and a higher rate of malnutrition identified by RFH-NPT might be a particular characteristic of patients with cirrhosis. In patients with cirrhosis, fluid retention can conceal true changes in body weight. Because malnutrition develops gradually, SGA questions on unintended weight loss spanning more than a month may limit accurate evaluation. In addition, patient-reported information on diet, symptoms, and physical abilities may be affected by recall inaccuracies or subclinical encephalopathy reducing the reliability of SGA [10]. In a cohort of 315 cirrhotic patients evaluated for liver transplantation, a study found weak concordance between SGA categories and sarcopenia (assessed by CT-based skeletal muscle index, SMI). Specifically, among patients with sarcopenia, many were classified as SGA A/B (i.e., “well nourished” or only mildly/moderately malnourished), and 20% of SGA A patients had sarcopenia [65,66]. In line with prior work comparing nutritional tools in cirrhosis, studies have shown that combining RFH-NPT with objective anthropometric or functional measures such as MUAC, MAMC, or handgrip strength identifies malnutrition and/or sarcopenia more reliably than SGA alone, especially in presence of fluid overload or sarcopenia [41,66].

Regarding nutrition, vitamin D supplementation has been shown to improve frailty, muscle strength, and lean muscle mass [67]. Furthermore, vitamin D was found to be an essential modulator of inflammatory status, with an increased number of TH1cells in cirrhotic patients with low levels of 25 (OH) vitamin D. After supplementation, lower levels of IL-1β and IL-6 were observed, with improvement in working memory (46.7 ± 13 to 50 ± 11; *p*= 0.047) [68]. Vitamin D deficiency and insufficiency have been correlated with worsening of hepatic encephalopathy [69].

Folate insufficiency has been associated with the appearance and progression of a variety of liver diseases through mechanisms that imply methionine metabolism, DNA synthesis, and epigenetic modulation of gene expression [70,71]. Moreover, it leads to increased production of pro-inflammatory cytokines, altered lipid metabolism (elevated triglycerides, low high-density lipoprotein (HDL) levels), fat accumulation in hepatocytes, and fibrosis [71,72]. On the other hand, excessive folate levels can cover up a vitamin B12 deficiency and facilitate HCC appearance and development [73]. Furthermore, folic acid supplementation in decompensated cirrhosis was associated with higher 1-year survival and reduced rehospitalization within one year [74].

In recent years, there has been increased interest in evaluating the role of magnesium in chronic liver disease. It has been linked to anti-inflammatory and antifibrotic effects [75]. The pathophysiological mechanism implicated in chronic liver disease is multifactorial: it is related to Mg absorption, depletion, and elimination, further exacerbating liver damage through stimulation of an inflammatory response, altering energy metabolism, disrupting signaling pathways, and increasing collagen deposition [76,77,78]. Mg supplementation in chronic liver disease might be a potent therapeutic modality to combat the ongoing pathophysiological mechanisms.

A nutritional intervention should be implemented for every cirrhotic patient, as emphasized in a meta-analysis of 14 randomized trials involving 1437 patients. BCAA supplementation improves the handgrip strength test, and using home-based intensive nutrition therapy (HINT) and a high-calorie high-protein (HCHP) diet, the muscle strength can be increased (HCHP has a higher effect on HGST than HINT diet) [79]. Moreover, it has been shown that a sixteen-week controlled diet and 60 min/week of physical exercise were associated with lower BMI and lower portal pressure in overweight/obese cirrhotic patients [80]. A study from 2024 showed that nasogastric feeding in cirrhotic patients with malnutrition awaiting liver transplantation improved handgrip strength by 20% and a 2-fold increase in protein and energy intake was observed [81].

This study has several limitations. Our cohort included only a small number of patients, of whom a small percentage were female, so differences between males and females were not included. In addition, a significant proportion of patients had moderate or severe ascites and/or peripheral edema, which could influence the results of 6MWT. As the muscle quantity and quality were not evaluated for all patients, an evaluation of sarcopenic patients could not be included.

## 5. Conclusions

Cirrhosis has been linked with malnutrition and sarcopenia in recent years. The risk for malnourished cirrhotic patients is increasing with decompensation, associated complications, comorbidities, and also alcoholic etiology. A comprehensive evaluation for all cirrhotic patients is necessary, with specific therapeutic approaches implemented. Despite their clinical importance, the real-world assessment of nutritional status and QoL remains variable, with gaps in standardization, feasibility, and clinical applicability.

This study is a real-life evaluation of patients with liver cirrhosis referred to a tertiary center, revealing a low quality of life for the patients and a high prevalence of malnutrition.

## Figures and Tables

**Table 1:** The GLIM criteria for evaluation of malnutrition [6].

Phenotypic criteria	Weight loss (%)	>5% within the past 6 months >10% beyond 6 months.
	Low BMI (kg/m^2^)	<20 for <70 years <22 for >70 years
	Reduced muscle mass	Evaluated with body composition measuring techniques
Etiologic criteria	Reduced food intake/assimilation	≤50% > 1 week or any reduction for >2 weeks or any chronic GI condition that alters food assimilation/absorption
	Inflammation	Acute disease/injury or chronic disease-related

**Table 2:** Diagnostic criteria for sarcopenia according to the EWGSOP 2 criteria [13].

Diagnostic Criteria According to EWGSOP2	Diagnostic Indicators	Male	Female
Low muscle strength	Handgrip strength test	<27 kg	<16 kg
Low muscle quantity or quality	Skeletal muscle index (SMI)	<7 kg/m^2^	<5.5 kg/m^2^
Low physical performance	Gait speed	<0.8 m/s	

**Table 3:** Baseline characteristics of the studied group.

Parameter				Total
Age mean (STD)				62.36 ± 9.28
Cigarette smoking				33.96% (*n* = 18)
Etiology % (*n*)			Alcohol use	75.47% (*n* = 40)
			HBV	3.77% (*n* = 2)
			HBC	7.55% (*n* = 4)
			Mixt	7.55% (*n* = 4)
			Other	3.77% (*n* = 2)
			MASLD	1.89% (*n* = 1)
Child–Pugh score % (*n*)			A	30.20% (*n* = 16)
			B	43.44% (*n* = 23)
			C	26.40% (*n* = 14)
Child–Pugh score (points)				8.57 ± 2.26
MELD (mean ± SD)				15.67 ± 7.068
MELD-Na (mean ± SD)				18.02 ± 7.86
MELD 3.0				18.30 ± 8.01
Grade of ascites % (*n*)			0	37.74% (*n* = 20)
			1	20.75% (*n* = 11)
			2	13.21% (*n* = 7)
			3	28.03% (*n* = 15)
Peripheric edema				50.94% (*n* = 27)
Hepatic encephalopathy				5.7% (*n* = 3)
DM				24.53% (*n* = 13)
CRD			without	92.45% (*n* = 49)
			with	7.55% (*n* = 4)
Antisecretory drugs				37.7% (*n* = 20)
Antibiotherapy				62.30% (*n* = 33)
Diuretics use				67.92% (*n* = 36)
Beta blockers	Propranolol			43.39% (*n* = 23)
	Carvedilol			37.7% (*n* = 20)
Weight (kg) mean ± SD				81.49 ± 17.85
Height (m) mean ± SD				1.73 ± 0.17
BMI (kg/m^2^) mean ± SD				27.61 ± 4.73
BMI correction ^1^ (kg/m^2^) mean ± SD:				25.06 ± 5.63
− Underweight % (*n*)				11.32% (*n* = 6)
− Normal weight % (*n*)				35.84% (*n* = 19)
− Overweight % (*n*)				52.83% (*n* = 28)
Laboratory (median, percentile 25; 75)				
Leukocytes				6.14 (5.15; 9.17)
Hemoglobin (g/dL)				11.70 (9.95; 13.05)
Platelets				116,000 (77,250; 156,500)
ALT (U/L)				29.00 (15.00; 52.00)
AST (U/L)				51.00 (33.00; 85.00)
GGT (U/L)				82.00 (31.50; 191.00)
Total bilirubin (mg/dL)				1.70 (0.93; 3.55)
Urea (mg/dL)				33.10 (26.50; 48.15)
Creatinine (mg/dL)				0.74 (0.53; 1.32)
PCR				14.70 (6.55; 25.23)
INR				1.36 (1.24; 1.75)
Albumin (g/dL)				3.00 (2.55; 3.80)
Cholesterol (mg/dL)				122.00 (90.50; 159.00)
Triglycerides (mg/dL)				78.50 (90.50; 159.00)
Sodium				136.00 (132.50; 138.00)
Potassium				4.02 (3.80; 4.34)
Magnesium				1.73 (1.57; 1.87)
Iron				83.45 (51.25; 132.10)
Folic acid				5.80 (3.78; 7.83)
B12 levels				570.00 (344.50; 1137.50)
Vitamin D				11.90 (9.05; 18.90)
Ammonia				84.70 (65.55; 109.22)
Questionnaires and tests				
HGS (mean ± SD)				15.96 ± 6.82
6MWT (%) mean ± SD				50.48% ± 31.48
6MWT (m) mean ± SD				246.41 ± 155.77
Gait speed (m/s) mean ± SD				0.68 ± 0.43
RFH-NPT		Low		15.09%(*n* = 8)
		Medium		35.84%(*n* = 19)
		High		49.05% (*n* = 26)
SGA		A		66.03% (*n* = 35)
		B		20.75% (*n* = 11)
		C		13.20% (*n* = 7)

^1^ BMI correction was applied depending on the grade of ascites and the presence of peripheric edema. HBV—Hepatitis B Virus, HBC—Hepatitis C Virus, SGA—subjective global assessment, RFH-NPT—Royal Free Hospital-Nutritional Prioritizing Tool, HGS—handgrip strength test, DM—diabetes mellitus, CRD—chronic kidney disease, ALT—alanine transaminase, AST—aspartate aminotransferase, GGT—gamma-glutamyl transferase.

**Table 4:** The reliability of the CLDQ and the correlation with the Child–Pugh, MELD, MELD-Na, and MELD 3.0 scores.

CLDQ Domains	Mean ± SD	Cronbach’s Alpha	Child–Pugh Score Value-Pearson	*p* Value	MELD Pearson Correlation Coefficient	*p* Value	MELD-Na Pearson Correlation Coefficient	*p* Value	MELD 3.0	*p* Value
Abdominal symptoms	4.70 ± 1.66	0.844	−0.266	0.054	−0.007	0.959	−0.054	0.701	−0.090	0.521
Fatigue	4.26 ± 1.63	0.795	−0.420	0.02 *	−0.324	0.018 *	−0.334	0.014 *	−0.341	0.013 *
Systemic symptoms	4.95 ± 1.19	0.820	−0.371	0.006 *	−0.272	0.049 *	−0.296	0.031 *	−0.322	0.019 *
Activity	4.79 ± 1.54	0.842	−0.333	0.015 *	−0.156	0.265	−0.209	0.133	−0.238	0.087
Emotional function	4.74 ± 1.37	0.829	−0.123	0.379	−0.166	0.235	−0.165	0.239	−0.195	0.162
Worry	4.61 ± 2.02	0.847	−0.347	0.011 *	−0.133	0.344	−0.157	0.261	−0.197	0.158
Overall CLDQ	4.67 ± 1.24	0.854	−0.372	0.006 *	−0.228	0.101	−0.254	0.066	−0.289	0.036 *

* *p* < 0.05.

**Table 5:** Distribution of CLDQ score/sub-scores according to disease severity evaluated with Child–Pugh Score.

Child–Pugh Score	Abdominal Symptoms	Fatigue	Systemic Symptoms	Activity	Emotional Function	Worry	Average CLDQ
A	5.20 ± 1.66	5.06 ± 1.56	5.60 ± 1.07	5.43 ± 1.49	4.86 ± 1.38	5.50 ± 1.25	5.27 ± 1.11
B	4.85 ± 1.27	4.47 ± 1.27	4.95 ± 1.01	4.94 ± 1.26	4.88 ± 1.33	4.54 ± 2.28	4.77 ± 0.96
C	3.85 ± 2.00	3.00 ± 1.57	4.21 ± 1.20	3.78 ± 1.60	4.34 ± 1.45	3.68 ± 1.93	3.81 ± 1.38
*p* value	0.069	0.001 *	0.004 *	0.009 *	0.464	0.045 *	0.003 *

* *p* < 0.05.

**Table 6:** Distribution of the patients depending on the severity of cirrhosis and RFH-NPT and SGA tests.

Test		Child–Pugh Score			MELD (Mean ± SD)	MELD-Na (Mean ± SD)	MELD 3.0 (Mean ± SD)
		A	B	C			
RFH-NPT	Low	22.64% (*n* = 12)	9.43% (*n* = 5)	3.77% (*n* = 2)	12.79 ± 5.56	14.05 ± 6.74	13.74 ± 6.58
	Moderate	7.54% (*n* = 4)	5.66% (*n* = 3)	1.88% (*n* = 1)	15.63 ± 7.62	17.63 ± 7.96	19.00 ± 7.38
	High	0% (*n* = 0)	28.30% (*n* = 15)	20.75% (*n* = 11)	17.81 ± 7.37	21.04 ± 7.52	21.42 ± 7.80
SGA	A	28.30% (*n* = 15)	26.41% (*n* = 14)	11.32% (*n* = 6)	14.71 ± 7.26	16.74 ± 8.17	17.06 ± 8.38
	B	1.88% (*n* = 1)	7.54% (*n* = 4)	11.32% (*n* = 6)	17.91 ± 7.12	20.27 ± 7.03	20.82 ± 6.75
	C	0% (*n* = 0)	9.43% (*n* = 5)	3.77% (*n* = 2)	17.00 ± 5.77	20.86 ± 6.82	20.57 ± 7.46

## Data Availability

The data presented in this study are available on request from the corresponding author.

## Abbreviations

QoL	Quality of life
BMI	Body mass index
EWGSOP 2	The revised European Working Group on Sarcopenia in Older People guidelines
GLIM	The Global Leadership Initiative on Malnutrition
CT	Computer tomography
DEXA	Dual-energy X-ray absorptiometry
6MWT	6 min walk test
CLDQ	The Chronic Liver Disease Questionnaire
SGA	The subjective global assessment
HGS	Handgrip strength test
HDL	High-density lipoprotein
HE	Hepatic encephalopathy
DM	Diabetes mellitus
CKD	Chronic kidney disease
ALT	Alanine transaminase
AST	Aspartate aminotransferase
GGT	Gamma-glutamyl transferase
HINT	Home-based intensive nutrition therapy
HCHP	High-calorie high-protein diet
SBP	Spontaneous bacterial peritonitis
RFH-NPT	The Royal Free Hospital-Nutritional Prioritizing Tool
HCC	Hepatocellular carcinoma
SMI	Skeletal muscle index
